# Pre-Planning the Surgical Target for Optimal Implant Positioning in Robotic-Assisted Total Knee Arthroplasty

**DOI:** 10.3390/bioengineering10050543

**Published:** 2023-04-28

**Authors:** Periklis Tzanetis, René Fluit, Kevin de Souza, Seonaid Robertson, Bart Koopman, Nico Verdonschot

**Affiliations:** 1 Department of Biomechanical Engineering, University of Twente, 7522 LW Enschede, The Netherlands; 2 Faculty of Science and Engineering, University of Groningen, 9747 AG Groningen, The Netherlands; 3 Orthopaedic Research Laboratory, Radboud Institute for Health Sciences, Radboud University Medical Center, 6525 GA Nijmegen, The Netherlands; 4 Stryker, Manchester M20 2HJ, UK

**Keywords:** pre-operative planning, robotic-assisted total knee arthroplasty, pre-diseased knee, musculoskeletal modeling, optimal implant position

## Abstract

Robotic-assisted total knee arthroplasty can attain highly accurate implantation. However, the target for optimal positioning of the components remains debatable. One of the proposed targets is to recreate the functional status of the pre-diseased knee. The aim of this study was to demonstrate the feasibility of reproducing the pre-diseased kinematics and strains of the ligaments and, subsequently, use that information to optimize the position of the femoral and tibial components. For this purpose, we segmented the pre-operative computed tomography of one patient with knee osteoarthritis using an image-based statistical shape model and built a patient-specific musculoskeletal model of the pre-diseased knee. This model was initially implanted with a cruciate-retaining total knee system according to mechanical alignment principles; and an optimization algorithm was then configured seeking the optimal position of the components that minimized the root-mean-square deviation between the pre-diseased and post-operative kinematics and/or ligament strains. With concurrent optimization for kinematics and ligament strains, we managed to reduce the deviations from 2.4 ± 1.4 mm (translations) and 2.7 ± 0.7° (rotations) with mechanical alignment to 1.1 ± 0.5 mm and 1.1 ± 0.6°, and the strains from 6.5% to lower than 3.2% over all the ligaments. These findings confirm that adjusting the implant position from the initial plan allows for a closer match with the pre-diseased biomechanical situation, which can be utilized to optimize the pre-planning of robotic-assisted surgery.

## 1. Introduction

The positioning of the implant in total knee arthroplasty (TKA) has been identified as one of the most important surgical choices determining the post-operative functional status of the knee [1]. Adequate three-dimensional implantation of the femoral and tibial components facilitates appropriate tensioning of the soft-tissue envelope and balancing throughout the arc of knee flexion; concomitantly, implant malpositioning may induce abnormal tensions in the ligamentous structures that encompass the tibiofemoral articulation, leading to kinematic deviations and complications with regard to joint stability [2,3]. Previous studies report that internal malrotation of the tibial component tightens the medial collateral ligament and increases asymmetrically the lateral femoral rollback in deep flexion [4,5]. Robotic-assisted surgery has been introduced to accurately position the components and reduce the outliers [6], and assist in achieving symmetrically balanced flexion and extension gaps [7]. The pre-planning of the robotic-assisted operation utilizes pre-operative computed tomography (CT) of the lower extremity to construct the geometrical model of the patient’s knee and selected bony landmarks, which facilitates identifying the optimal size of the components and target position. These anatomical renderings guide the intra-operative bone resections within the pre-planned spatial boundaries and subsequent implant positioning, thus limiting cortical bone and periarticular soft-tissue injuries [8,9]. In a cadaveric study, Hamp et al. demonstrated that robotic-assisted TKA (RA-TKA) improved the precision of bone resection and achieved highly accurate positioning in the three planes compared to manual intervention [10]. The attained accuracy has been reported to be within 1 mm and 1° of the pre-operative plan [11]. Despite the high degree of accuracy, it remains debated whether utilizing robotic-assisted surgery translates into improved functional outcomes [12]. This raises the question of what the optimal position of the components should be to ensure optimal post-operative joint function. There are various positioning strategies that have been proposed to attain a functional and balanced knee joint after surgery [13]. Traditionally, mechanical alignment (MA) has been used for implant placement since it could be performed with traditional imaging and conventional surgical instruments. MA strives for a neutral post-operative mechanical axis [14]. This approach, however, may be undesirable for patients with constitutional varus [15]. Alternatively, others have suggested a kinematically aligned implantation that recreates the joint line obliquity and tibiofemoral kinematics of the native knee [13,16], although reports display unbalanced soft tissues in kinematically aligned knees with pre-operative deformities due to unequal mediolateral flexion gaps [17,18]. More recently, the functional alignment technique, as an adaptive step from either mechanical [19] or kinematic [20] alignment, has been proposed to maintain a balance in the mediolateral soft-tissue envelope. With the advent of RA-TKA, orthopaedic surgeons can define more post-operative targets, tailoring the operation to the individual patient. One of the proposed targets in personalized surgery is to recreate the functional aspects of the knee before the onset of the disease, referred here as the pre-diseased knee. Hence, targeting the reproduction of the healthy knee, kinematic patterns, and its accompanying strain patterns of the surrounding soft tissues.

The aim of this study was first to investigate deviations in knee kinematics and ligament strains between the pre-diseased and corresponding mechanically implanted knee and, second, to demonstrate the feasibility of reproducing the pre-diseased functional profiles, testing different surgical targets, by optimizing the implant position from the initial mechanical implantation. We hypothesized that fine-tuning the positioning parameters of the individual components would allow to achieve a closer match between the pre-diseased knee conditions and the post-operative knee kinematic and ligament strain profiles than MA.

## 2. Materials and Methods

**Figure 1:**
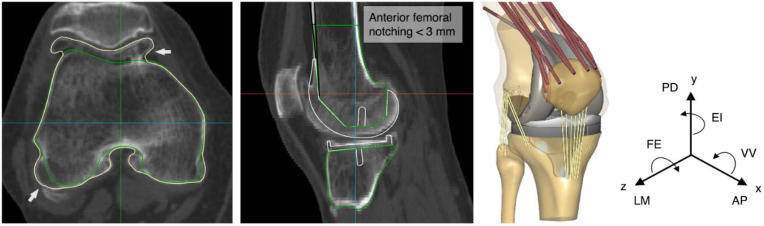
From left to right, axial view of the patient’s computed tomography (CT) scan indicating the osteophyte (white arrows, yellow line) and osteophyte-free femoral boundaries (green line); sagittal view of the mechanically aligning (MA) virtual planning; and the patient-specific musculoskeletal total knee arthroplasty (TKA) model. Nomenclature: AP, anterior-posterior; LM, lateral-medial; PD, proximal-distal; FE, flexion-extension; EI, external-internal; VV, varus-valgus.

We used a previously developed musculoskeletal knee model of a cadaveric specimen as a reference for the subsequent personalization to the patient-specific data [21]. This model was implemented in the AnyBody Modeling System (AnyBody Technology A/S, Aalborg, Denmark). Structurally, the model comprises the thigh, shank, and patella and simulates a knee extension movement from 60° to 0° under unloaded conditions, with only the gravitational force acting along the y-axis of the system (Figure 1). The flexion angle of the unconstrained tibiofemoral joint is controlled using a simple motion driver about the z-axis of joint rotation while the remaining degrees of freedom equilibrate quasi-statically under the effect of quadriceps muscles, ligaments, and contact forces. The following ligaments were included in the model to maintain stability: anterior cruciate ligament (ACL), posterior cruciate ligament (PCL), deep medial collateral ligament (dMCL), superficial medial collateral ligament (sMCL), lateral collateral ligament (LCL), anterolateral ligament (ALL), oblique popliteal ligament (OPL), and posterior capsule (PC). The mechanical parameters assigned to the individual ligament bundles are provided elsewhere [21,22]. The imaging data used in the present study were part of the knee functional flexion axis dataset [23]. The abstracted data contained the pre-operative CT scan of the lower extremity of a single patient with severe knee osteoarthritis who underwent primary TKA using the Stryker Knee Navigation system (Stryker, Kalamazoo, MI, USA). We segmented the pre-operative CT image using a statistical shape model provided by Imorphics (Stryker, Manchester, UK) that was trained to recreate the osteophyte-free femoral and tibial geometric boundaries. The osteophyte volume detection algorithm has been previously described [21,24,25]. The resolution of the training CT images was about 0.3–0.6 mm within the axial/transverse (XY) slice and 1.0–1.5 mm between slices (Z). The statistical shape model used to perform the auto-segmentation was not limited, however, by the between-slice resolution, and has been shown to achieve an accuracy of approximately 0.13 mm in all three dimensions [26]. The reconstructed geometries were used to morph the topology of the cadaveric reference bones to the corresponding patient-specific bones without osteophytes and, subsequently, the attachments of the ligaments, employing a non-linear morphing technique [22]. This constituted the pre-diseased knee. The anatomical frames for the femur, tibia, and patella were established using bony landmarks digitized at the hip, knee, and ankle segments, following a pre-defined convention [27]. A magnetic resonance image-based statistical shape model was used to calculate the thickness of the cartilage from the segmented bone surfaces following a validated method for cartilage segmentation [28].

The patient-specific model was initially implanted according to MA principles with the Triathlon single-radius cruciate-retaining (CR) total knee system (Stryker Orthopaedics, Mahwah, NJ, USA) (Figure 1). A size-5 femoral component was chosen to ensure congruence with the femoral condyles and avoid excessive mediolateral overhang that might cause an irritation to the surrounding soft-tissues. The thickness of the tibial polyethylene insert was 9 mm, which provided a good trade-off between the tibial resection depth and the collateral ligament strains [29]. The patella was not resurfaced. Knee kinematics, specifically anterior-posterior (AP), lateral-medial (LM), and proximal-distal (PD) tibial translations, as well as tibial external-internal (EI) rotation, and varus-valgus (VV), were calculated with both the pre-diseased and post-operative models during knee extension against gravity from 60° to 0° according to Grood and Suntay’s joint coordinate system definition [30]. Strains of the ligaments were reported throughout the same range of motion. Differences between the pre-diseased and post-operative model predictions were quantified in terms of root-mean-square deviation (RMSD) over the range of motion.

An optimization scheme was then employed, seeking the optimal position of the components that minimize the differences between the pre-diseased and post-operative kinematics (surgical target 1) or ligament strains (surgical target 2). This optimization problem was solved by minimizing a quadratic objective function (Equation (1)), subject to the boundaries defining the optimal position, which was adjusted for each surgical target.

(1) $$\underset{x\in R^{n}}{\min}\sum_{i=1}^{m}w_{i}\sqrt{\frac{1}{t}\sum_{\theta =0}^{\theta_{end}}({{y_{{implanted}_{i,\theta }}\left(x\right)-y}_{{pre\text{-}\mathrm{diseased}}_{i,\theta }})}^{2}}+P\;lb<x<ub$$

where x indicates the positional parameter of the femoral and tibial component with lb and ub denoting its lower and upper boundaries, respectively (Table 1). The boundary conditions are relative to the initially estimated MA femoral and tibial component positions and are comparable to those used in similar studies [31,32,33]. The width of the FE boundaries of the femoral component was narrower to avoid anterior notching of the femoral cortex greater than 3 mm, which can be detrimental to the surgical outcomes [34,35]. The translational position of the tibial component remained unchanged to ensure convergence of the optimization problem. Further, n indicates the dimension of the solution space equal to the number of x positional parameters (n=9), m is the number of variables included in the objective function, $w_{i}$ is the weighting factor for the $i^{th}$ variable (default 1), t indicates the number of simulation time steps between 0 and $\theta_{end}$ that was set equal to 60, $\theta_{end}$ denotes the terminal knee flexion angle equal to 60°, and $y_{i,\theta }$ denotes the $i^{th}$ tibiofemoral kinematic variable or the strain of the $i^{th}$ ligament at knee flexion angle θ. P denotes the applied penalties; a penalty of $10^{3}$ was added to the objective function if the force-dependent-kinematics solver applied to the model generated a residual force in the knee joint that exceeded the specified error tolerance of 5 N, ensuring that only dynamically consistent simulations were taken into consideration [36].

In the objective function for surgical targets 1 and 2, the kinematic variables and ligament strains were weighted equally. To account for the important role of the collateral ligaments in controlling the joint stability [37,38], we re-executed the optimization process, assigning an eight-fold increased weighting to the medial and lateral collateral ligaments in Equation (1) (surgical target 3). This choice aimed to approximate more closely the pre-diseased functioning behavior of those ligaments during the entire range of motion. Since stretching the ligaments beyond their pre-diseased capacity can accumulate damage [39], which might also be related to pain, we imposed further constraints on the objective function of ligament strains to ensure that the strains remain within the estimated peak of the pre-diseased curve (surgical target 4). Specifically, a penalty factor of $10^{3}$ was applied to the objective function if the strain value at any given flexion angle exceeded the peak of the pre-diseased strain. We ultimately modified the objective function to concurrently optimize for kinematics and ligament strains in a unified objective (surgical target 5) that maintains both kinematic and strain deviations close to the pre-diseased state. In this process, the objective function was formulated as the summation of two separate functions, one corresponding to kinematics and the other to ligament strains; $w_{i}$ was chosen such that the kinematic and strain errors contributed equally to the objective function. The covariance matrix adaptation evolution strategy (CMA-ES) [40] was applied to find the global optimal implant position for each of the five surgical targets. CMA-ES is an evolutionary algorithm in which new candidate solutions are generated by selecting and varying the fittest parent candidates in each subsequent generation. This process generated a solution space for implant positioning, from which we selected the solutions with the minimum deviation from MA to ensure that the selected optima not only follow the objective function requirements but they are also applicable in the clinical setting [41]. The optimization routine was performed using a 64-core 2.9 GHz processor, which required approximately 32 h to determine the optimal placement of the implant.

## 3. Results

**Figure 2:**
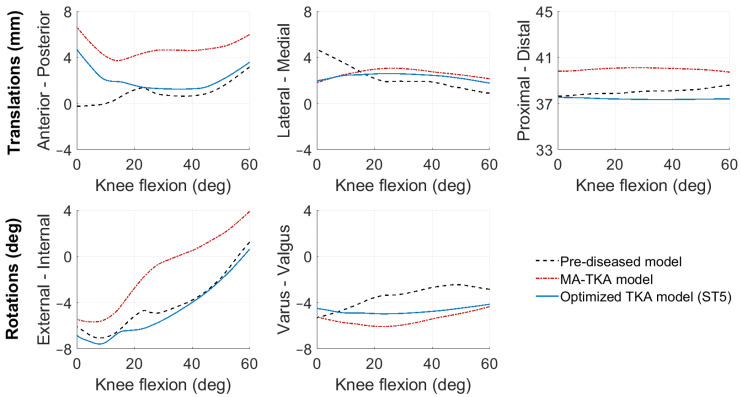
Tibiofemoral kinematics predicted by the pre-diseased, MA, and optimized TKA models during a knee extension movement from 60° to 0°. The optimized TKA model represents the results after concurrent optimization for both kinematics and ligament strains (surgical target 5). Positive curve values denote anterior, lateral, and proximal translations, and external and varus rotations. Nomenclature: ST5, surgical target 5.

**Figure 3:**
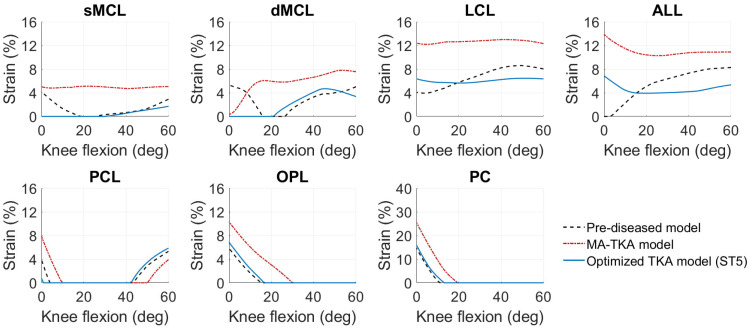
Ligament strains predicted by the pre-diseased, MA, and optimized TKA models during a knee extension movement from 60° to 0°. The optimized TKA model represents the results after concurrent optimization for both kinematics and ligament strains (surgical target 5). Strain is expressed as a percent strain. Nomenclature: sMCL, superficial medial collateral ligament; dMCL, deep medial collateral ligament; LCL, lateral collateral ligament; ALL, anterolateral ligament; PCL, posterior cruciate ligament; OPL, oblique popliteal ligament; PC, posterior capsule.

**Figure 4:**
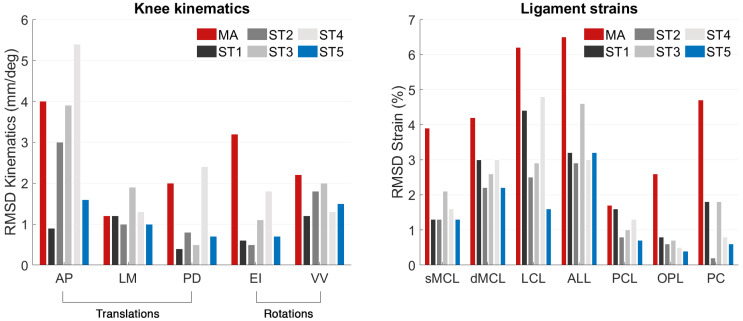
Root-mean-square deviations (RMSD) in knee kinematics and ligament strains between the pre-diseased and post-TKA models for MA and each of the examined surgical targets. AP, LM, and PD translations are expressed in mm, while EI and VV rotations in degrees. Nomenclature: ST1, surgical target 1; ST2, surgical target 2; ST3, surgical target 3; ST4, surgical target 4; ST5, surgical target 5.

**Figure 5:**
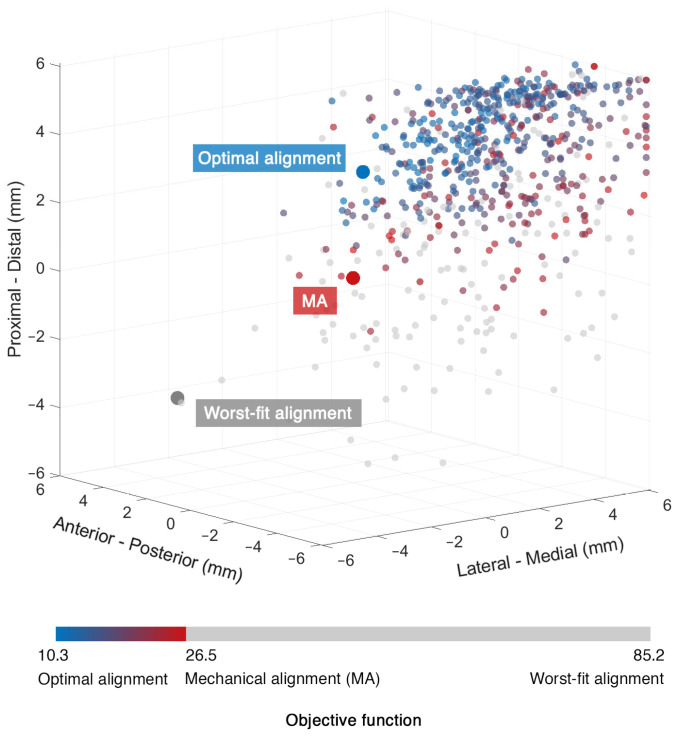
Illustrative case of the translational alignment of the femoral component, showing the three-dimensional (3D) distribution of the solution space from the concurrent optimization for kinematics and ligament strains (surgical target 5). Each sphere in the 3D space represents a unique solution colored based on the response of the objective function. The color scheme ranges from blue, representing potential optimal solutions, to red, representing solutions closest to MA. Gray spheres indicate less optimal solutions. Positive values denote posterior, medial, and proximal translations.

**Figure 6:**
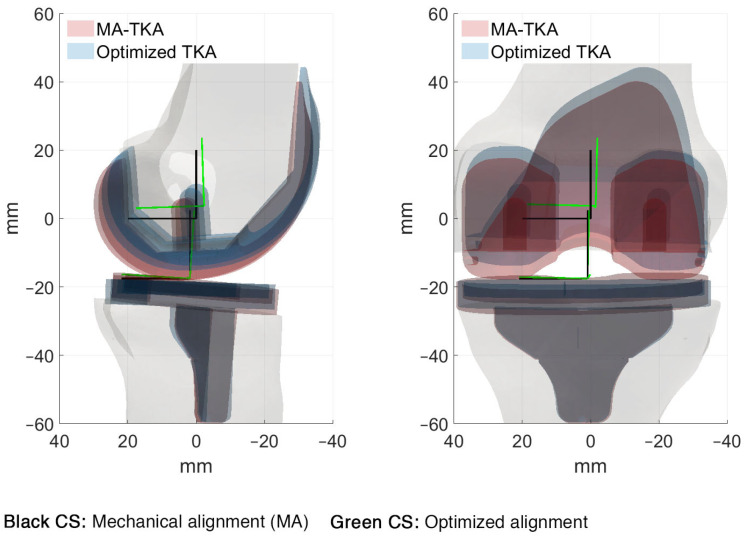
Visualization of the implant in situ following bone resection according to MA and optimized implant alignment, along with the coordinate systems (CS) of the femoral and tibial components, to concurrently reproduce the pre-diseased kinematics and ligament strains (surgical target 5). The interface gap between the anterior flange of the optimally positioned implant and the bone was not observed, as depicted in this image.

The MA-TKA model predicted knee kinematics with an RMSD of 2.4 ± 1.4 mm and 2.7 ± 0.7° compared to the pre-diseased knee. Ligament strains deviated up to 4.2% in the medial collateral (sMCL, dMCL), 6.2% and 6.5% in the lateral collateral (LCL) and anterolateral (ALL) ligamentous structures, respectively, and up to 4.7% in the posterior structures (PCL, OPL, PC). With the optimized TKA model, we found an RMSD of 0.8 ± 0.4 mm and 0.9 ± 0.4° and strain deviations up to 4.4% over all the ligaments when optimizing only for kinematics (surgical target 1). When solely focusing on optimizing the ligaments (surgical target 2), strains exhibited an RMSD of up to 2.2% in the medial collateral, 2.5% and 2.9% in the lateral collateral and anterolateral structures, and up to 0.8% in the posterior structures; the kinematic deviations were slightly larger than those found with surgical target 1: 1.6 ± 1.2 mm and 1.1 ± 0.9°. Following the weighting adjustment of the collateral ligaments (surgical target 3), we observed strain deviations lower than 2.9% in the collateral ligaments, 4.6% in the anterolateral, and 1.8% in the posterior structures. By further constraining the strains of the ligaments during the entire range of motion to their corresponding pre-diseased peak value (surgical target 4), we recorded deviations up to 4.8% in the medial and lateral collateral ligaments and up to 1.3% in the posterior ligamentous structures. Concurrent optimization for both kinematics and ligament strains resulted in an RMSD of 1.1 ± 0.5 mm and 1.1 ± 0.6° compared to the pre-diseased state. Figure 2 shows the kinematic curves with the pre-diseased, MA, and optimized TKA models in comparison. Strains exhibited deviations lower than 2.2% in the sMCL and dMCL, 1.6% and 3.2% in the LCL and ALL, respectively, and lower than 0.7% in the PCL, OPL, and PC (Figure 3). Figure 4 summarizes the RMSD in the kinematic variables and strains of the individual ligaments between the pre-diseased and TKA models for MA and each of the five surgical targets. The positioning changes of the femoral and tibial components required to resemble the pre-diseased profiles, according to the selected optimality criterion, are reported in Table 2. Figure 5 provides an illustrative case of the translational alignment of the femoral component, displaying the spatial distribution of the solution space towards surgical target 5, and Figure 6 further visualizes the optimized alignment of the components in situ referenced to MA.

## 4. Discussion

This study investigated differences in kinematics and ligament strains, comparing a mechanically implanted knee with its corresponding pre-diseased state and, subsequently, determined the feasibility of restoring the functional status of the pre-diseased knee by optimizing the implant position. We demonstrated that fine-tuning the position of the femoral and tibial components from the initial MA plan allows for a closer match between the pre-diseased and post-TKA profiles. The optimal position of the components was variable and dependent on the optimality criterion.

MA of the knee implant generally affected the ligament strains and, consequently, the tibiofemoral kinematics in a clinically relevant manner. The LCL exhibited a near isometric behavior over the flexion range, with strain deviations between 4.3% and 8.3% compared to the pre-diseased situation (Figure 3). Previous in vivo studies suggest that strain deviations from the native knee should remain lower than 5% to prevent structural damage of the collateral ligaments [39,42] and, eventually, knee pain. The consistently higher strains observed in the LCL are likely to be related to the increased tibial external rotation (Figure 4), as this ligament resists rotational movement. The function of the ALL was clearly distinct, displaying an opposite trend from 0° to 30° compared to the pre-diseased strain, which could be attributed to the different configuration of the implanted articulating surface. The predicted straining of the ALL fibers could also explain the increased anterior translation (Figure 4) of the tibia during the entire range of motion [43]. On the medial side, the sMCL and dMCL strains after implantation were relatively consistent but higher, which consecutively resulted in increased valgus rotation (Figure 4). On the posterior ligamentous structures, the strain patterns of the PCL, OPL, and PC after MA-TKA were comparable to the pre-diseased curves and corroborate earlier findings [44]; peak strains were observed in extension, exhibiting an approximately 50% increase compared to the pre-diseased values, which cannot be disregarded and may potentially result in a tendency of post-operative extension deficit [45]. These strain magnitudes are still within the ultimate strain region [46,47].

The optimization routine for each of the five criteria identified a broad area of potential positioning solutions with minimal variations in the value of their objective function relative to the final optimal solution (Figure 5). With the optimized TKA model for kinematics, we managed to reduce the deviations by 66.7% for translations (RMSD<1.2 mm) and rotations (RMSD<1.2°) (Figure 4) relative to the pre-diseased knee. These results align with those of Dejtiar et al. [31], who reproduced the native kinematics with an average RMSD of 1.5 ± 0.9 mm and 2.9 ± 2.9° from MA-TKA by training a model-based artificial neural network. However, the authors did not account for the large rotational variability, reporting deviations greater than 4.0° in EI rotation with the optimally implanted model. In our study, the required positioning changes to reproduce the pre-diseased kinematics were within ±4 mm and ±5° from MA (Table 2). Of all the parameters that define the spatial positioning of the implant, the slope of the tibial component had the greatest effect. Marra et al. [48] demonstrated that increasing the posterior tibial slope up to 9° from neutral, referenced from the center of the tibial plateau, is not detrimental to the post-operative knee function. Surgeons should, however, carefully consider the native tibial slope of the patient when deciding on the appropriate level of slope correction, as excessive slope increase may affect the overall stability of the knee. The pre-diseased tibial slope in the present study was about 5°, as measured on the pre-operative CT image. It is also worth discussing that optimizing for kinematics results in a concomitant adjustment of the ligaments, indicating strain deviations within 0.8–4.4%, which are clinically acceptable [39]. Optimizing solely for the ligament strains resulted in a 57.2% average reduction in the dMCL and sMCL, 59.7% and 55.4% in the LCL and ALL, respectively, and 75.2% on average in the PCL, OPL, and PC. These outcomes are consistent with those of Quilez et al. [33], who accurately emulated the pre-operative ligament elongations using a model-based response surface methodology. The authors reported optimal implant position within ±5 mm and ±3° from MA. Similarly, in our study, the required position changes to reproduce the pre-diseased strains were in the range of ±4 mm and ±3°, peaking at 3.6 mm distal translation of the femoral and 2.8° internal rotation of the tibial component (Table 2). These positioning adjustments are unlikely to compromise the function of the implant after TKA [49]. Focusing further on the collateral ligaments yielded marginal alterations, yet maintaining the collateral ligament strain deviations below 3% (Figure 4). In addition, we were able to position the components such that the strains remain below the pre-diseased peak values (RMSD<4.8%) by modifying the implant position within the same pre-defined ranges. Nevertheless, we observed a near total slackening of the medial collateral ligaments throughout the entire range of motion, and therefore, these results should be interpreted with caution. Concurrently optimizing for kinematics and ligament strains allowed to reduce the translational (RMSD<1.6 mm) and rotational (RMSD<1.5°) deviations by 54.2% and 59.3%, respectively, and keep the strain deviations within 0.7–3.2% compared to the pre-diseased knee (Figure 4); this range is well below the damage threshold of 5% as previously described [39,50]. It appears that only the sMCL, dMCL, and ALL could not be reproduced in extension (Figure 3), which could be due to the shape of the implant rather than its position. With a non-patient-specific implant, we expect a compromise between positioning the components and achieving soft-tissue balance. Proximal shifting of the femoral component by 3.7 mm increased the joint gap, which eventually resulted in overall lower strains compared to the MA state. The slope of the tibial component also had a considerable impact, in accordance with an earlier study [33], which, however, suggested a tibial slope up to 15° to restore the pre-operative kinematics, which may not be clinically realistic. The required changes in implant position were larger than the accuracy attained in robotic-assisted surgery, except for the external rotation of the femoral component, which still falls within the achievable range of accuracy [10,11].

This simulation study is important as it precisely assesses the effects of various surgical optimization criteria, including kinematics and strains in the tibiofemoral ligaments, which is not achievable with cadaveric experiments. It will therefore contribute to further enhance utilization of robotic-assisted surgery, as with the methods described in this paper, the robotic system can be fed with a specific target. One of the major strengths of this study was the statistical shape model-based methodology employed to recreate the osteophyte-free bones, which enabled us to construct the geometrically patient-specific model of the knee before the onset of osteophytes. This approach can potentially overcome an important limitation of cadaveric studies, in which treatments are based on specimens that hardly reflect the anatomy of the healthy and/or osteoarthritic knee.

This study had several limitations. First, the musculoskeletal model, although state-of-the-art, has clear limiting factors: the model predictions could not be validated against experimental measurements; a comparable model [22] underwent validation using fluoroscopic data and predicted AP and PD translational errors in the order of the observed deviations (Figure 4), reiterating the importance of an in vivo tracking of joint mechanics in response to the changes in implant position. Additionally, the menisci were not modeled in the pre-diseased knee, which may have induced kinematic alterations in the posterior and mediolateral direction across the tibiofemoral joint [51]. The reported changes in the position of the components refer to an unloaded knee extension movement. Although we believe that kinematic and strain patterns similar to physiological in an unloaded state may also hold true under loaded conditions, this assumption remains to be proven. Finally, we disregarded the functioning of the patella relative to variations in the position of the femoral flange. Further research should investigate the effect of changes in the femoral component position on the patellofemoral joint; improper positioning of the femoral component can contribute to patellar maltracking and, eventually, anterior knee pain, which is one of the most common complications reported by patients after TKA [52]. Another limitation of this study was that we did not consider changes in the mechanical properties of the soft tissues, such as their stiffness and reference strain, owing to the progression of osteoarthritis. The present findings pertain solely to a single patient implanted with a size-5 CR-TKA. It would be interesting to apply the same optimization technique to a population-based cohort of TKA patients and assess different component sizes or patient-specific implant designs.

In summary, mechanically aligning the knee implant resulted in clinically relevant differences in the ligament strains and consequent kinematics compared to the pre-diseased status. These differences could be reduced by optimizing the position of the femoral and tibial components starting from MA. The required adjustments in the position of the components were variable and dependent on the surgical target. Since different targets have varying functional consequences, it remains to be determined which target leads to the best possible clinical outcomes. The proposed methodology establishes a quantitative framework that can assist orthopaedic surgeons in optimizing the pre-planning of robotic-assisted TKA, which strives to recreate the pre-diseased functional status of the knee, including the strains of the ligamentous structures and the tibiofemoral kinematics.

## Figures and Tables

**Table 1:** Implant positional parameters involved in the optimization routine.

Implant Positional Parameters	Boundary Conditions Relative to MA		Components
	Upper Bound	Lower Bound	
**Translations (mm)**			
AP	−6	+6	Femoral
LM	−6	+6	Femoral
PD	−6	+6	Femoral
**Rotations (°)**			
FE	−3	+3	Femoral, Tibial ^1^
EI	−6	+6	Femoral, Tibial
VV	−6	+6	Femoral, Tibial

^1^ The FE boundaries of the tibial component ranged from −6° to 6°. The translational position of the tibial component in the AP, LM, and PD directions remained unmodified. Positive values in the specified ranges denote posterior, medial, and proximal translations, and flexion, internal, and varus rotations of the components.

**Table 2:** Component positioning changes to achieve the examined surgical targets.

Surgical Target/ Components	Translations (mm)			Rotations (°)		
	AP	LM	PD	FE	EI	VV
**Surgical target 1**						
Femoral	−2.8	−0.2	3.9	−1.4	−0.2	−1.2
Tibial	-	-	-	5.2	2.8	2.2
**Surgical target 2**						
Femoral	−1.7	−1.8	3.6	−1.5	−1.6	−1.5
Tibial	-	-	-	0.9	2.8	−1.9
**Surgical target 3**						
Femoral	−1.1	0.1	2.3	0.9	−1.8	−0.4
Tibial	-	-	-	−1.8	−1.5	3.2
**Surgical target 4**						
Femoral	−2.4	−0.8	3.6	−0.6	−0.3	1.6
Tibial	-	-	-	−2.8	−0.2	2.6
**Surgical target 5**						
Femoral	−2.3	−1.5	3.7	−1.9	−0.5	−1.4
Tibial	-	-	-	3.6	2.3	−1.1

Surgical target 1: knee kinematics; surgical target 2: ligament strains; surgical target 3: ligament strains with increased weighting in the collateral ligaments; surgical target 4: ligament strains constrained to the pre-diseased peak value; surgical target 5: combined knee kinematics and ligament strains. Positive values denote posterior, medial, and proximal translations, and flexion, internal, and varus rotations.

## Data Availability

Not applicable.
